# Reflecting Digital Transformations in Public Health Curricula

**DOI:** 10.3389/phrs.2025.1608237

**Published:** 2025-03-12

**Authors:** Rok Hrzic, Stefan Buttigieg, Brian Li Han Wong, Anabelle Macedo Silva, Karl F. Conyard, Mary Codd, Patty Kostkova, Nienke M. Schutte, Robin van Kessel

**Affiliations:** ^1^ Department of International Health, Care and Public Health Research Institute–CAPHRI, Maastricht University, Maastricht, Netherlands; ^2^ Digital Public Health Task Force, Association of Schools of Public Health in the European Region (ASPHER), Brussels, Belgium; ^3^ Ministry for Health and Active Ageing, Valletta, Malta; ^4^ Institute of Studies in Collective Health (IESC), of the Federal University of Rio de Janeiro (UFRJ) Public Collective Health Institute, Federal University of Rio de Janeiro, Rio de Janario, Brazil; ^5^ MPRJ Ministério Público do Estado do Rio de Janeiro, State Public Attorney’s Office, Rio de Janeiro, Brazil; ^6^ LSCDiPH BIPS/UniBremen Leibniz Science Campus on Digital Public Health, BIPS/Bremen’s Epidemiology Institute, University of Bremen, Bremen, Germany; ^7^ School of Public Health, Physiotherapy and Sports Science, University College Dublin, Dublin, Ireland; ^8^ University of Medicine and Health Science, The Royal College of Surgeons in Ireland, Dublin, Ireland; ^9^ ASPHER Core Curriculum Programme, Association of Schools of Public Health in the European Region (ASPHER), Brussels, Belgium; ^10^ ASPHER Executive Board, Association of Schools of Public Health in the European Region (ASPHER), Brussels, Belgium; ^11^ UCL Centre for Digital Public Health in Emergencies (dPHE), University College London, London, United Kingdom; ^12^ Innovation in Health Information Systems Unit, Sciensano, Brussels, Belgium; ^13^ LSE Health, Department of Health Policy, London School of Economics and Political Science, London, United Kingdom

**Keywords:** public health education, digital health, curriculum development, digital competence, digital transformations

## Introduction

Digital technologies promise greater personalisation and precision in public health services, automation of repetitive tasks, and more efficient use of existing resources through rapid management and analysis of big data sets. To seize this opportunity, the public health workforce needs to become sufficiently competent to navigate these novel digital technologies and understand how to apply them across the spectrum of essential public health functions. With few exceptions, however, digital skills are not yet systematically incorporated into public health curricula.

## The Promise and Peril of Digital Transformations in Public Health

Digital transformation in public health is “a complex and multifaceted process that is disruptive and fundamentally changes the culture, operational models, and goals of public health services, centred on the health needs of the public.” [1] Critically, this includes not only the digitisation of existing processes and patient pathways but also devising entirely new ways of working, bringing about a cultural transformation [2].

The widespread adoption of smartphones, wearable technology, and social media platforms since the 2000s was seized as new opportunities for public health interventions, while the COVID-19 pandemic accelerated the uptake of digital technologies within the health sector [3, 4]. Legislation like the European Health Data Space regulation is creating increasingly sophisticated and technically advanced health information systems that require skilled professionals to manage efficiently and amplify the potential benefits of digital transformations [5]. On the other hand, the emergence of social media as a dominant source of health (mis)information, increasing concern about the mental health impacts of social media, and the growing role of “Big Tech” in public health also create new challenges that most public health professionals are ill-equipped to handle promptly and comprehensively [6]. On top of that, the emergence of a range of artificial intelligence technologies could redesign and positively augment central tenets of health systems and healthcare delivery while simultaneously posing a potential risk to public and population health through the spread of misinformation and reinforcement of health-harming behaviours [2]. To navigate the complexities of digital transformations in public health, there is an urgent need to upskill the public health workforce.

## Existing Guidance on Relevant Competencies

At the time of publication, there are no generally agreed-upon competency frameworks focused on digital transformations in public health. However, sufficient relevant evidence is available to start an initial redesign of public health curricula in anticipation of more comprehensive guidance. For instance, we can learn from competency frameworks aimed at healthcare providers. A recent review of educational frameworks identified 28 digital health competency domains, including basic information technology literacy, health information management, digital communication, ethical, legal, or regulatory requirements, and data privacy and security [7].

Ongoing global and Europe-wide initiatives will improve the theoretical and practical basis for training the (public) health workforce. Building on its experience with infodemic management training, the WHO convened a Digital Health Competency Framework Committee in 2023 to develop a framework outlining competencies for digital health policymakers, programme planners or managers, health practitioners, and the general public. Another example is the EU-funded BeWell project (bewell-project.eu), which developed a strategy for developing digital and green skills in healthcare and an overview of relevant training programmes [8].

Currently available resources (e.g., the WHO-ITU Digital Health Platform Handbook) [9] are geared towards helping professionals navigate and steer digital transformations in health organisations and services. These resources are not explicitly aimed at directing public health workforce development, risking the oversight of public health-specific factors. Nevertheless, they can offer a promising starting point for the augmentation and redesigning of public health curricula to include materials on digital transformations.

## Recommendations for Curriculum Redesign

Based on a comprehensive synthesis and expert consensus of priority areas in the digital determinants of health [2], we identified and described the competency domains for undergraduate and general graduate public health education that are widely accepted as critical to public health in the digital era (Table 1). However, the key challenge is implementing new competency domains in public health curricula that balance well-established public health competencies with the new competencies required by digital transformations. To support institutions and educators in navigating this challenge, we provide four guiding principles.

**TABLE 1:** Themes of competence relevant to digital transformations in public health.

Theme	Definition	Example priority educational components
Using digital tools	Provide essential public health functions using digital tools (hardware and software) and navigate emerging disruptive technologies like artificial intelligence	Understand public health information systems Understand infostructures and digital health platforms with a focus on public health functions
Digital health literacy and digital determinants of health	Digital health literacy is the ability to seek, understand, evaluate, and use digital health information and tools to make informed health decisions and effectively engage with healthcare systems	Understand the concept of digital health literacy Understand the role of the digital determinants of health
Management and leadership skills applied to digital transformations in health	Deep understanding of the relevant skills necessary to implement pragmatic digital transformation in Public Health, including agile project management, change management, and behaviour change	Understand and apply digital project management approaches, including Waterfall, Agile, and others Understand digital behavioural change strategies, including behavioural design thinking Understand change management in the context of digital transformations Develop the case for digital transformation, focusing on the business case and financial planning
Health data collection and analysis	Collate, evaluate, and analyse health data from existing and emerging sources (e.g., electronic health records, social media, wearables, etc.)	Understand data transfer agreements Assess data quality Apply various approaches to modelling big data, including machine learning
Health data management and governance	Organise, manage, and govern health data efficiently following the FAIR principles to support research efficiency	Understand FAIR principles and implement international guidelines Understand regulatory frameworks for data government and management Develop a health data management plan Perform a data maturity assessment Understand and apply basic cybersecurity practices
Ethics and regulation of digital transformations in society	Understand and apply ethical frameworks to navigate data privacy, digital and data equity, and other relevant challenges	Understand the role of national, EU, and global regulation in protecting the digital dimension of fundamental rights Understand the general principles of the GDPR and the legal basis for justifying the use of personal data
Infosphere and spread of information over digital networks	Understand the spread of information across digital networks and apply these insights in designing effective communication strategies, including on social media	Measure and monitor the impact of infodemics during health emergencies Detect and study the spread and impact of infodemics Apply interventions that mitigate an infodemic and protect a population from its harmful effects Evaluate infodemic interventions Develop and apply strategies to strengthen the resilience of individuals and communities to infodemics Develop, adapt, and apply tools for the management of infodemics
The safe, ethical and sustainable use of artificial intelligence (AI) in health	The safe, ethical, and sustainable use of AI in health involves understanding and applying principles that ensure AI technologies are used responsibly in public health	Understand various AI technologies and their applications in public health settings Critically appraise AI solutions used in a public health setting Understand the regulatory and ethical frameworks supporting AI in healthcare and public health Understand the importance of sustainability and equity in AI adoption, including concerns about its environmental impact, bias, and accessibility Understand the use of artificial intelligence in public health research

Firstly, it is important to integrate relevant digital competencies throughout the curriculum rather than exclusively in standalone courses on digital transformations - especially in light of the ubiquitous nature of digitalisation [2]. This allows staff and students to contend with the challenges of digital transformation in the relevant context and avoids counterproductive pigeonholing of digital skills [10]. For example, emerging sources of health data can be studied in the context of an existing course on epidemiology, and digital communication skills can be integrated into existing courses on health promotion. Secondly, integrating digital competencies in organisational management and leadership skills is necessary to reflect digital transformations as a fundamental process of change in how public health services are delivered. Thirdly, fostering literacy in managing organisations and leading organisational change is essential. These themes are already highlighted in the WHO-ASPHER Competency Framework for the Public Health Workforce in the European Region. Some schools already provide relevant content in their core programmes. This experience, supplemented by the work of specialist groups such as the ASPHER Task Force on Digital Transformation in Public Health (DiPH), has contributed to the proposed curricular content in the ASPHER Core Curriculum for Public Health (www.ccp.aspher.org), a current and ongoing dynamic effort to define and harmonise PH curricula in support of the attainment of PH competencies. This also covers the potential to upskill the existing public health workforce through executive training and other forms of continuous professional development.

Finally, it is vital to create an interdisciplinary academic community with a deep understanding of information technology and data engineering as well as public health disciplines. Digital transformations in public health require that the public health workforce be prepared to work closely with other professionals in and outside of the health sector, including employees of social media companies, software developers, data engineers and scientists [2]. This requires a bidirectional transition to an interdisciplinary curriculum. In one direction, public health education needs to foster digital skills in students with backgrounds in medicine or the health sciences. In the other direction, public health schools need to provide training in the principles of public health for information technology and engineering students interested in digital public health. To achieve this, public health schools will need to work collaboratively with faculties of computer science and engineering.

## Group Members of the ASPHER Task Force on Digital Public Health Includes

Stefan Buttigieg, Clara Bermúdez-Tamayo, Mary Codd, Karl F. Conyard, Keren Dopelt, Mariusz Duplaga, Paula Sofia Herrera Espejel, Giovanna Failla, Magda Fonseca, Michal Grivna, Stephanie Hoffmann, Rok Hrzic, Alexandre Jaborska, Jaime Jimenez, Robin van Kessel, Patty Kostkova, Mirjana Kujundžić Tiljak, Szczepan Jakubowski, Anabelle Macedo Silva, Laura Maaß, Ana Cecilia Quiroga Gutierrez, Anna Odone, Gaetano Privitera, Nienke M. Schutte, Mariam Shokralla, Azhar Talal, Joost Verbeek, and Brian Li Han Wong.
